# Author Correction: Spatial rearrangement of the *Streptomyces venezuelae* linear chromosome during sporogenic development

**DOI:** 10.1038/s41467-021-25897-6

**Published:** 2021-09-16

**Authors:** Marcin J. Szafran, Tomasz Małecki, Agnieszka Strzałka, Katarzyna Pawlikiewicz, Julia Duława, Anna Zarek, Agnieszka Kois-Ostrowska, Kim C. Findlay, Tung B. K. Le, Dagmara Jakimowicz

**Affiliations:** 1 Faculty of Biotechnology, University of Wrocław, Wrocław, Poland; 2 The John Innes Centre, Norwich Research Park, Norwich, UK

**Keywords:** Chromosomes, Bacterial development, Bacterial genetics, Cellular microbiology

Correction to: *Nature Communications* 10.1038/s41467-021-25461-2, published online 1 September 2021.

The original version of this Article omitted the following from the Acknowledgements:

‘and BBSRC (BBS/E/J/000PR9791) for funding’.

This has now been corrected in both the PDF and HTML versions of the Article.

