# Supplementary material for: Spatial rearrangement of the Streptomyces venezuelae linear chromosome during sporogenic development
Source: Nat Commun. 2021 Sep 1;12:5222. doi: 10.1038/s41467-021-25461-2 (PMC8410768; doi:10.1038/s41467-021-25461-2)
Supplement: Supplementary file 1 — Supplementary Information [file 41467_2021_25461_MOESM1_ESM.pdf]

A

Sample	Time Point [h]	Replicate	Pairs Sequenced [mln]	Pairs mappable, unique and high quality [mln]	Pairs used for Hi-C matrix [mln]	Pairs used [%]
control_22 (no PFA)	22	Replicate 1	5,97	5,12	1,33	22,33%
wt22_1	22	Replicate 1	17,20	15,20	2,24	13,01%
wt22_2	22	Replicate 2	17,77	13,83	4,75	26,74%
parB22_1	22	Replicate 1	20,48	17,29	1,66	7,82%
parB22_2	22	Replicate 2	28,32	22,76	5,31	18,76%
smc22_1	22	Replicate 1	21,48	18,74	1,14	5,31%
smc22_2	22	Replicate2	16,32	13,60	2,69	16,52%
hupS22_1	22	Replicate 1	25,25	22,29	1,13	4,49%
hupS22_2	22	Replicate 2	17,75	15,00	0,96	5,40%
smc_hupS25_1	25	Replicate 1	17,65	14,47	3,00	17,04%
smc_hupS25_2	25	Replicate 2	13,82	11,00	1,34	8,22%
parA22_1	22	Replicate 1	21,76	16,89	6,05	27,81%
parA22_2	22	Replicate 2	23,46	18,24	5,55	23,67%
wt13_1	13	Replicate 1	20,75	16,66	2,85	13,72%
wt13_2	13	Replicate 2	21,17	17,00	5,72	27,03%
wt15_1	15	Replicate 1	22,89	18,61	3,72	16,25%
wt15_2	15	Replicate 2	16,82	12,82	3,70	22,02%
wt17_1	17	Replicate 1	25,56	20,38	6,13	23,98%
wt17_2	17	Replicate 2	23,12	18,86	4,65	20,13%
wt25_1	25	Replicate 1	15,96	13,10	1,90	11,85%
wt25_2	25	Replicate 2	13,78	11,33	1,96	14,26%

B

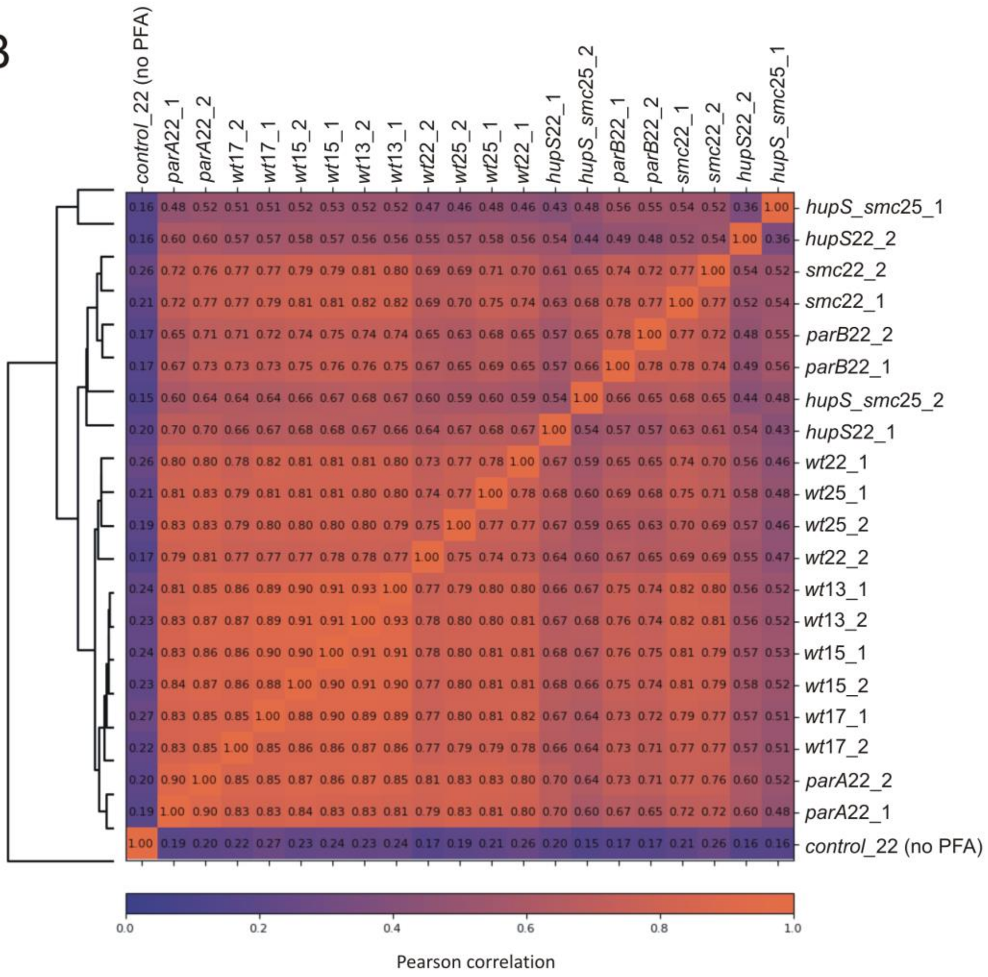

Figure S1

Supplementary Figure 1. Statistics and data reproducibility. (A) Summary of raw sequencing data used for Hi-C matrix preparation for particular time point. The table shows the number of paired reads for each sample (each with two biological replicates), the number of pairs mapped to the *S. venezuelae* chromosome (according to the *Bowtie2* settings, see Methods) as well as the number of pairs successfully filtered and used subsequently for Hi-C matrix construction (mln pairs and % of total pairs sequenced). **(B)** Pairwise correlation analysis of the normalized Hi-C matrices for particular time point performed with the *hicCorrelate* package (version Galaxy 3.6+galaxy0). The Hi-C matrices were grouped by their similarities based on the Pearson correlation coefficient. The values of the Pearson correlation coefficient are shown in the diagram. The wild type strain growing for 22 h and not treated with paraformaldehyde (no PFA, see Methods) served as a control.

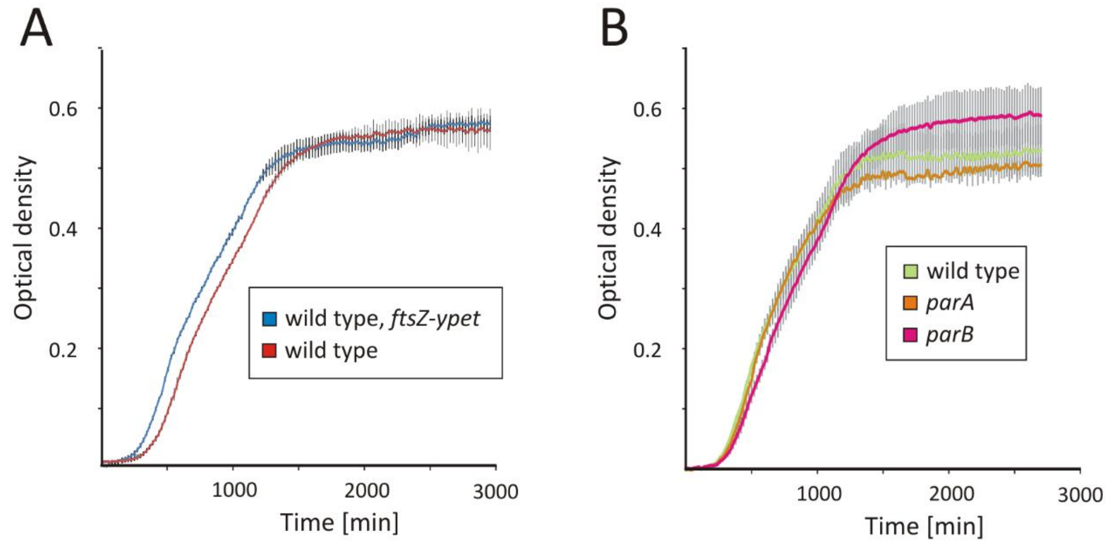

Figure S2

Supplementary Figure 2. The growth rate of *S. venezuelae* strains. (A) The growth of the *S. venezuelae* wild-type strain (red) and its *ftsZ-ypet* derivative (MD100, blue) in MYM medium quantified using a Bioscreen C instrument (optical density A_{600} measured in 20 min intervals). **(B)** The growth of the wild-type strain (green) and *parA* (orange) and *parB* (pink) mutant strains in MYM medium quantified using a Bioscreen C instrument. All analysed strains were *ftsZ-ypet* derivatives (MD100, MD011 and MD021). In A and B the colour lines and error bars correspond to the mean values and the standard deviations, respectively, calculated for each time point from 3 independent experimental replicates.

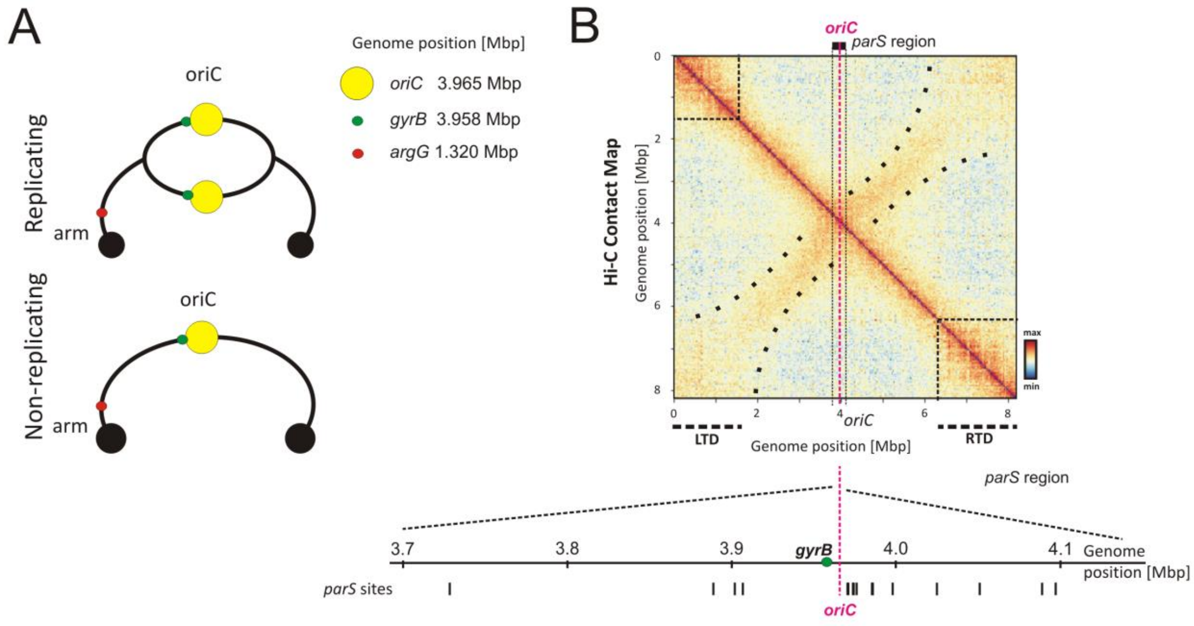

Figure S3

Supplementary Figure 3. The organization of the linear *S. venezuelae* chromosome. (A) Scheme of markers used for *oriC*/*arm* frequency analysis of DNA replication. The scheme shows the chromosomal position [Mbp] of the *oriC* region (yellow) and *gyrB* locus, which served as an *oriC* marker (green), as well as the marker of the left chromosomal arm, the *argG* locus (red). **(B)** The Hi-C contact map for the wild-type strain after 22 h of growth. The black dashed lines mark the positions of the left (LTD) and right (RTD) terminal domains as well as the boundaries of the contacts along the secondary diagonal axis. The *oriC* site (pink dashed line), *gyrB* loci (green dot) and the *parS* cluster (black) are marked on the Hi-C map (the exact positions of the *parS* sites and *oriC* are also shown below as black lines).

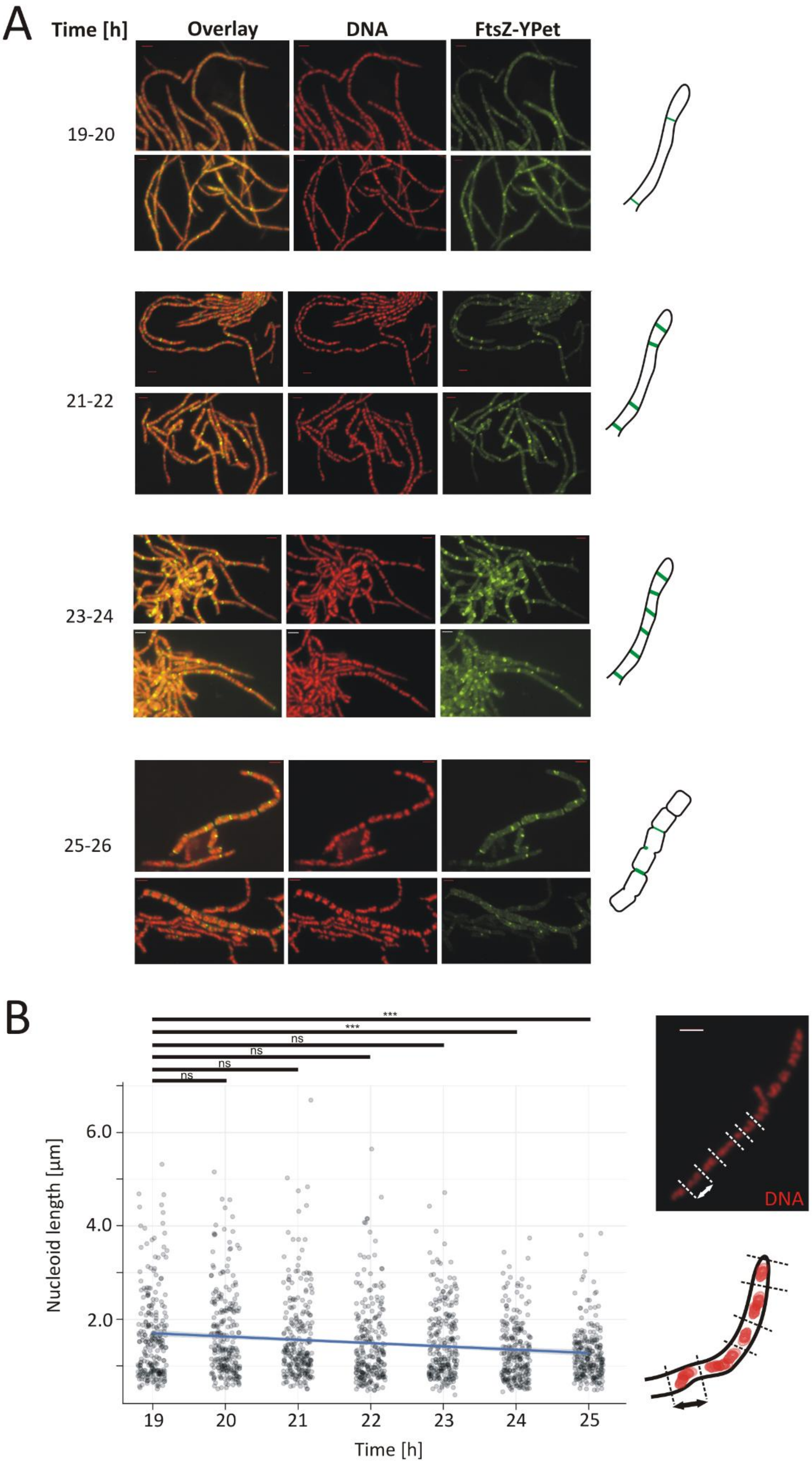
A
Time [h]
Overlay
DNA
FtsZ-YPet
19-20
21-22
23-24
25-26
B
***
***
ns
ns
ns
ns
Nucleoid length [µm]
6.0
4.0
2.0
19
20
21
22
23
24
25
Time [h]
DNA

Figure S4

Supplementary Figure 4. Formation of Z-ring ladders and nucleoid compaction during *S. venezuelae* sporulation. **(A)** Fluorescence microscopy images of the wild type strain (*ftsZ-ypet* derivative, MD100) hyphae fixed at different time points of culture (5 ml culture). The images show the visualization of nucleoids (DNA stained with 7-AAD) and the FtsZ-YPet signal. The schematic drawings on the right-hand side show the hyphae at particular time points of *S. venezuelae* sporogenic development. (**B**) Analysis of nucleoid compaction (n = 250 nucleoids) at the different time points of *S. venezuelae* growth. Nucleoid compaction was quantified as the distance (marked with a black arrow) between the centres of two DNA-free zones. The blue line corresponds to the linear model fitted to the collected data. The inset shows a representative picture showing an example of the data collection process. Scale bar - 2 µm. The statistical significance between strains determined by the Wilcoxon test (two-sided) is marked with asterisks: p-value ≤0.01 (**), ≤0.001 (***) and >0.05 (ns). p-values: 19h-20h: 0.999, 19h-21h: 0.995, 19h-22h: 0.272, 19h-23h: 0.529, 19h-24h: 5.27e-05, 19h-25h: 3.49e-07.

Principal Component Analysis (PCA1)

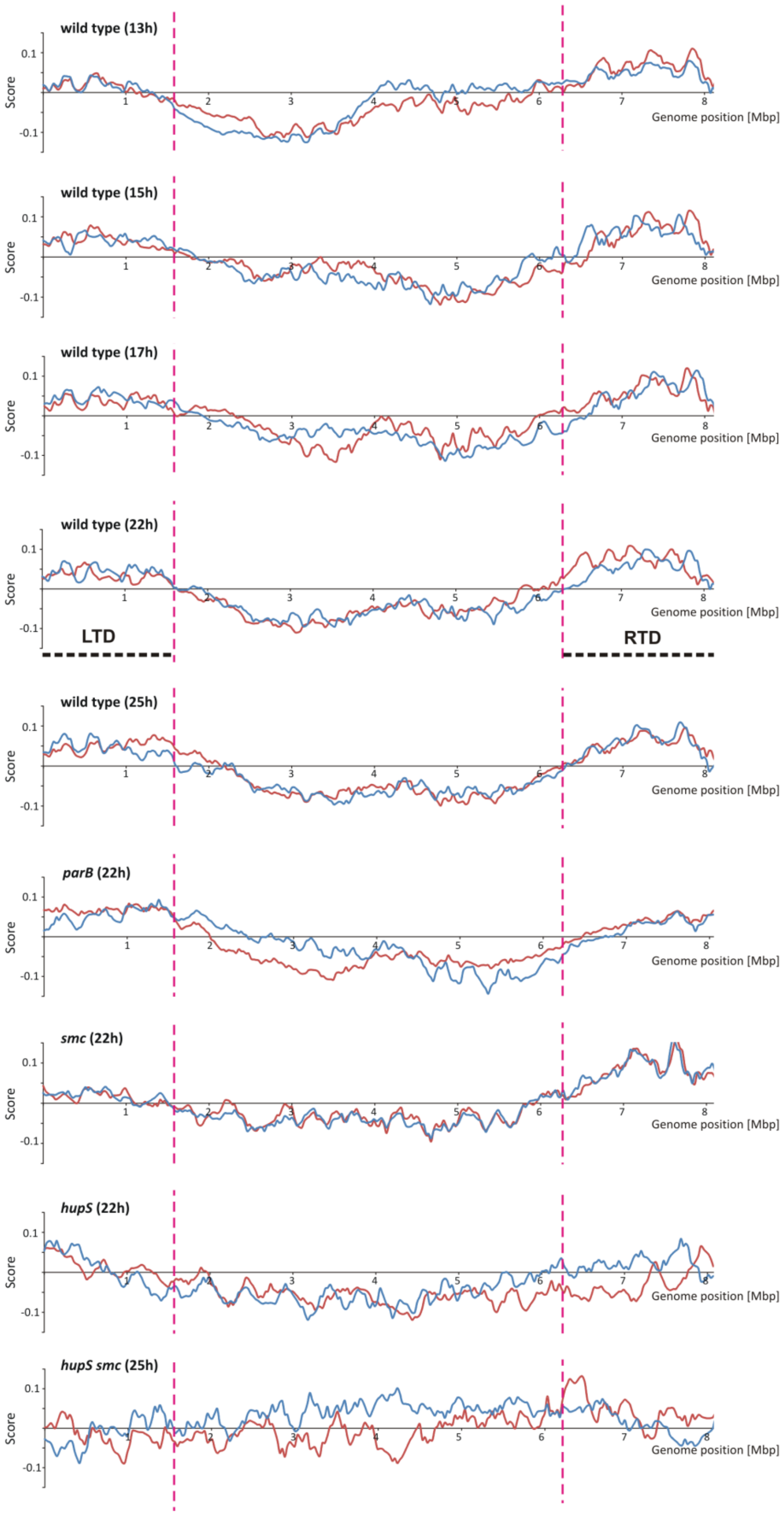

Figure S5

Supplementary Figure 5. Identification of distinct regions within the *S. venezuelae* chromosome. Principal component analysis (PCA1) of the corrected and normalized Hi-C matrices is shown in correlation with *S. venezuelae* genome position [Mbp]. Red and blue lines correspond to the independent biological replicates. The positions of the left (LTD) and right (RTD) terminal domains identified in contact maps (see also Fig. 1B) of the wild-type strain (*ftsZ-ypet* derivative, MD100) grown for 22 h (5 ml cultures) are marked with black dotted lines. The determined boundaries of the RTD and LTD domains for 22 h of growth were subsequently overlayed (pink dashed lines) on the PCA1 diagrams plotted for all tested time points or various *S. venezuelae* mutants.

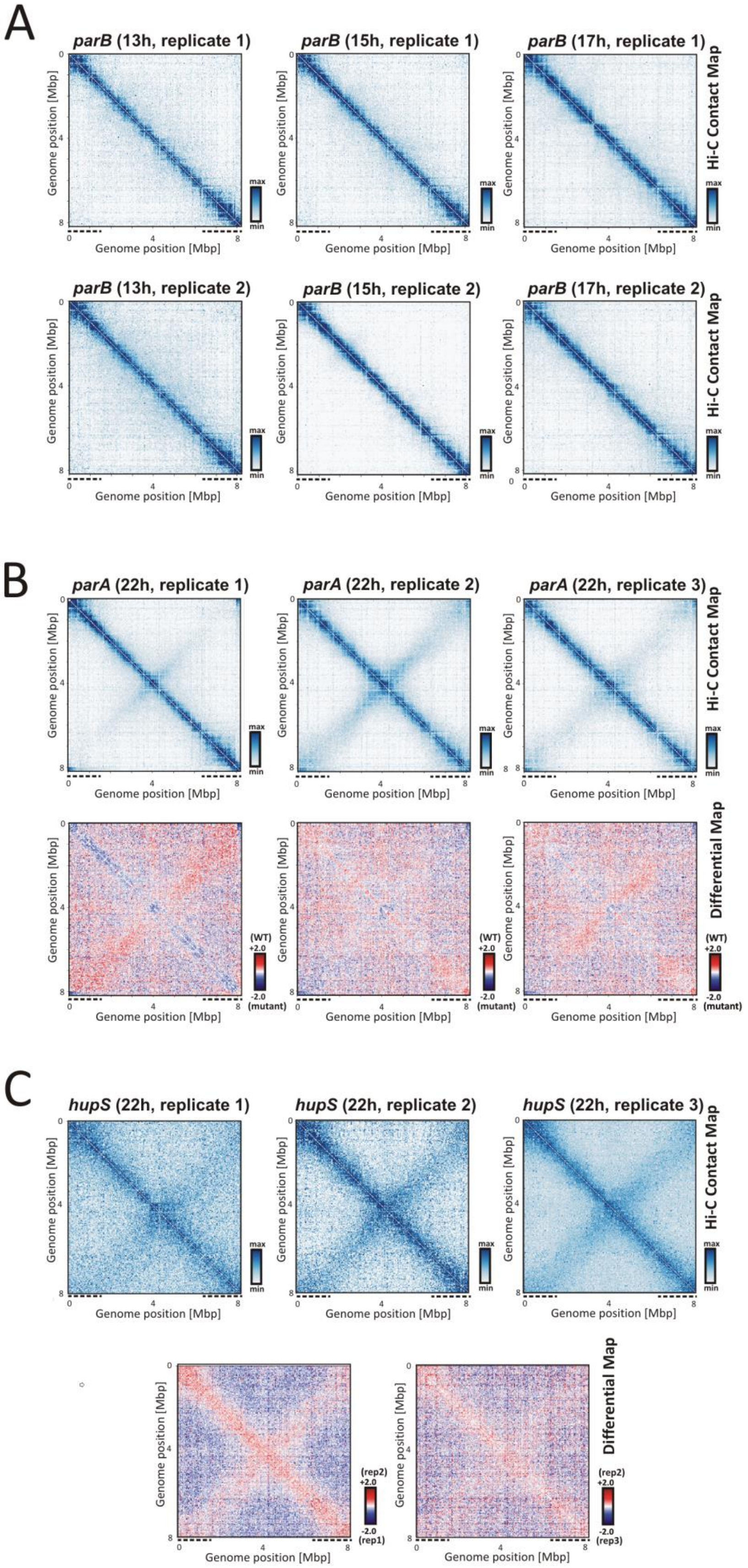
A
parB (13h, replicate 1)
parB (15h, replicate 1)
parB (17h, replicate 1)
parB (13h, replicate 2)
parB (15h, replicate 2)
parB (17h, replicate 2)
Hi-C Contact Map
Genome position [Mbp]
max
min
B
parA (22h, replicate 1)
parA (22h, replicate 2)
parA (22h, replicate 3)
Differential Map
(WT)
+2.0
-2.0
(mutant)
C
hupS (22h, replicate 1)
hupS (22h, replicate 2)
hupS (22h, replicate 3)
(rep2)
(rep1)
(rep3)

Figure S6

Supplementary Figure 6. Chromosome organization of *parA, parB* and *hupS* mutants. (A) The normalized Hi-C contact maps obtained for two biological repeats of the *parB* mutant (*ftsZ-ypet* derivative, MD021) at 13, 15, and 17 h of growth (5 ml culture). **(B)** Top panel: the normalized Hi-C contact maps obtained for three biological repeats of the *parA* mutant (*ftsZ-ypet* derivative, MD011) grown for 22 h (5 ml culture). Bottom panel: the differential contact maps in the logarithmic scale (log2) comparing the contact enrichment in the wild-type strain (red) versus the *parA* mutant (blue) are shown below each replicate. **(C)** Top panel: the normalized Hi-C contact maps obtained for three biological repeats of the *hupS* mutant (*ftsZ-ypet* derivative, TM005) grown for 22 h (5 ml culture). Bottom panel: the differential contact maps in the logarithmic scale (log2) comparing the contact enrichment in *hupS* mutant replicates 1 and 3 (blue) versus replicate 2 (red) are shown below the pair of biological replicates.

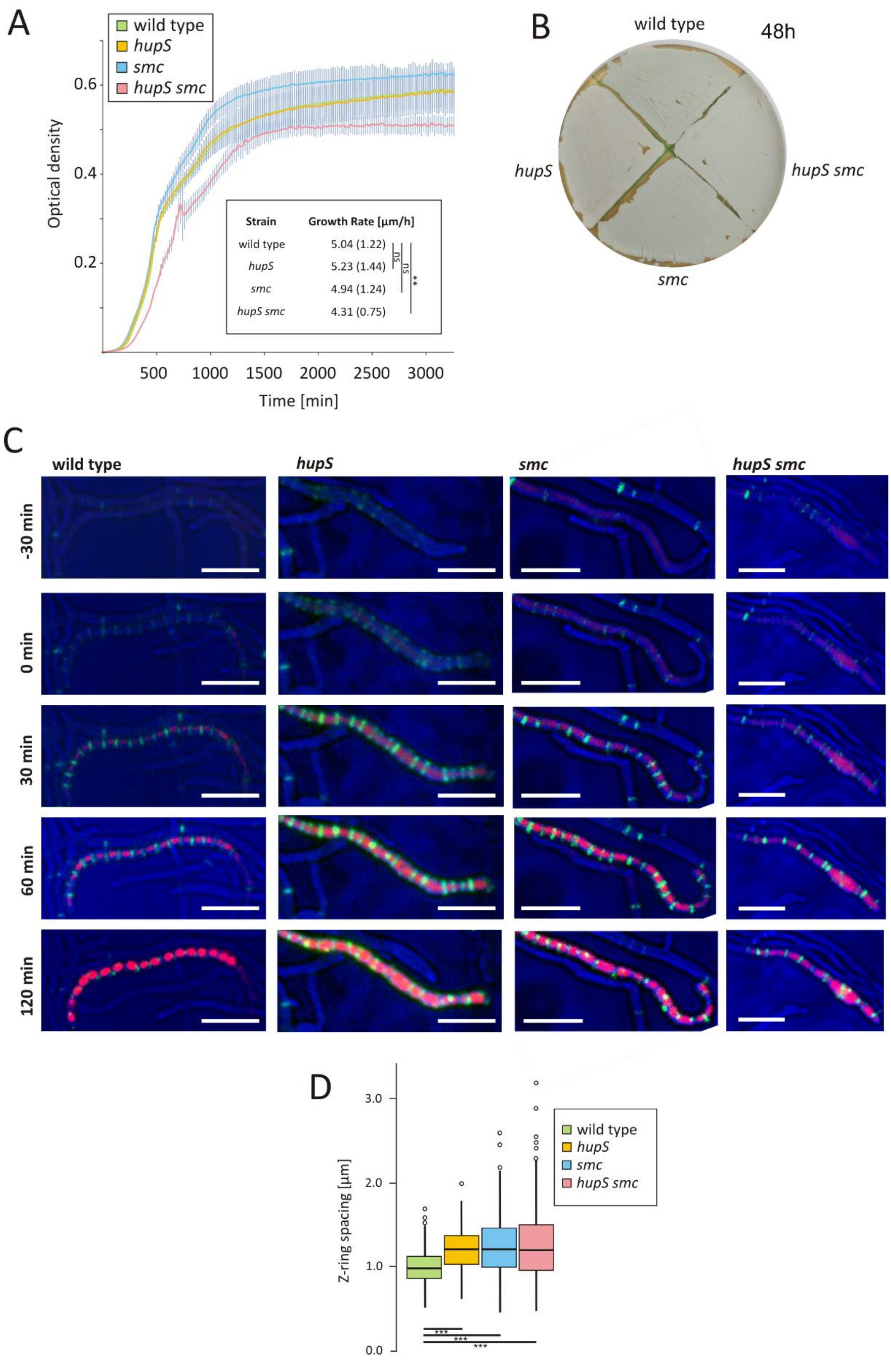
A
wild type
hupS
smc
hupS smc
Optical density
0.2
0.4
0.6
500
1000
1500
2000
2500
3000
Time [min]
Strain
Growth Rate [µm/h]
wild type 5.04 (1.22)
hupS 5.23 (1.44)
smc 4.94 (1.24)
hupS smc 4.31 (0.75)
ns
ns
**
B
wild type
48h
hupS
hupS smc
smc
C
wild type
hupS
smc
hupS smc
-30 min
0 min
30 min
60 min
120 min
D
Z-ring spacing [µm]
0.0
1.0
2.0
3.0
wild type
hupS
smc
hupS smc
***
***
***

Figure S7

Supplementary Figure 7. Detailed analysis of the phenotypic effects of *smc*, *hupS* and double *smc hupS* deletion. (A) The growth of the wild type (light green), *hupS* (yellow), *smc* (blue) and *hupS smc* (pink) double mutant (*ftsZ-ypet* derivatives, MD100, TM005, TM004, TM006) in MYM medium quantified using a Bioscreen C instrument. The colour lines and error bars correspond to the mean values and the standard deviations, respectively, calculated for each time point from 3 independent experimental replicates. The inset shows the average growth rate [µm/min] of a germ tube (50 hyphae of each strain) observed in the time-lapse experiment. Standard deviations are shown in brackets. The statistical significance between strains determined by a one-way anova with a Games-Howell post-hoc test (two-sided) is marked with asterisks: p value ≤0.05 (*), ≤0.01 (**) and ≤0.001 (***). p-values: wild type-*hupS*: 0.899, wild type-*smc*: 0.976, wild type-*hupS smc*: 0.003. **(B)** The growth of the wild type as well as *hupS*, *smc* and *hupS smc* double mutant analysed after 48h of growth on solid MYM medium (AKO200, TM010, TM003, respectively). **(C)** Time-lapse observations of the wild type as well as *hupS*, *smc* and *hupS smc* double mutant (*ftsZ-ypet*, *hupA-mCherry* derivatives, TM011, TM013, TM012 and TM014, respectively). Nucleoid condensation was visualized using mCherry-HupA fusion, whereas Z-rings were visualized using FtsZ-YPet fusion. The representative images (of more than 10 repetitions) show the hyphae 30 min before and up to 130 min after the growth arrest of the sporogenic compartment (time = 0 min). Scale bar: 5 µm. **(D)** Box plot analysis of the Z-ring spacing distribution. The distance between two Z-rings was measured during the sporulation of the wild type as well as *hupS*, *smc* and *hupS smc* double mutant (*ftsZ-ypet* derivatives, TM005, TM004, TM006, respectively). Boxplots show median with first and third quartile while the lower and upper "whiskers" extend to the value no further than 1.5 * IQR (interquartile range) from the “hinge”. The statistical significance between strains determined by Games-

Howell test (two-sided) is marked with asterisks: p value ≤0.05 (*), ≤0.01 (**) and ≤0.001 (***). p-values: wild type-*hupS*: <.0001, wild type-*smc*: <.0001, wild type-*hupS smc*: <.0001.

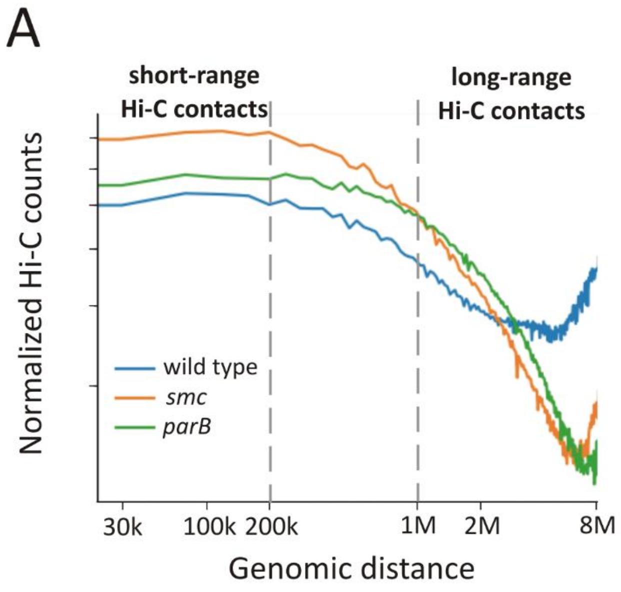

Figure S8

Supplementary Figure 8. Involvement of SMC and ParB in short- and long-range DNA contacts. (**A**) The normalized Hi-C contacts calculated using the *hiCPlotDistVsCounts* package (Galaxy Version 3.4.3.0) for the wild type strain (blue), *smc* (orange) and *parB* (green) mutants (*ftsZ-ypet* derivatives, MD100, TM004 and MD021, respectively). The X-axis shows the genomic position on a logarithmic scale. The short-range (below 200 kbp) and long-range Hi-C contacts (above 1 Mbp) are marked with dotted lines. (**B**) Analysis of the nucleoid area in the wild-type strain (n = 530), *smc* (n = 610) and *parB* (n = 176) mutants (*ftsZ*-derivatives, MD100, TM004 and MD021, respectively). Boxplots show the median with first and third quartiles while the lower and upper "whiskers" extend to the value no further than 1.5 * IQR (interquartile range) from the "hinge". The statistical significance between strains determined by the Games-Howell test (two-sided) is marked with asterisks: p value ≤0.05 (*), ≤0.01 (**) and ≤0.001 (***). p-values: wt-*smc*: <.0001, wt-*parB*: 0.0326.

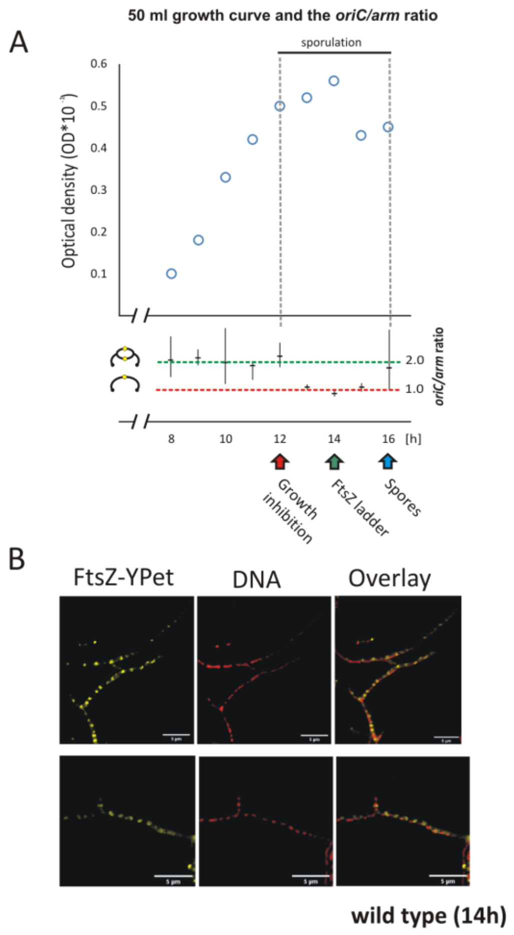

Figure S9

Supplementary Figure 9. Standardization of the *S. venezuelae* "ChIP-seq culture". (A) The growth curve under ChIP-seq culture conditions (50 ml cultures) performed in n = 1 experimental repeat. The critical time points of sporogenic development are marked with arrows: growth arrest (red), the appearance of FtsZ ladders (green) and the formation of spore chains (blue). The relative *oriC/arm* ratio is shown below the plot, with X axes corresponding to the main plot X axis. The *oriC/arm* ratio for chromosomal DNA isolated from 26 h culture at Hi-C conditions growth was set as 1.0. N = 3 independent experiments; mean *oriC/arm* values as well as calculated standard deviations ("whiskers") are shown on the diagram. **(B)** Examples fluorescence microscopy images (representative of 10 images) of

the wild type (*ftsZ-ypet* derivative MD100) hyphae fixed at 14h of growth (50 ml culture). The images show the visualization of nucleoids (DNA stained with 7AAD) and the FtsZ-YPet signal. Scale bar: 5 µm.

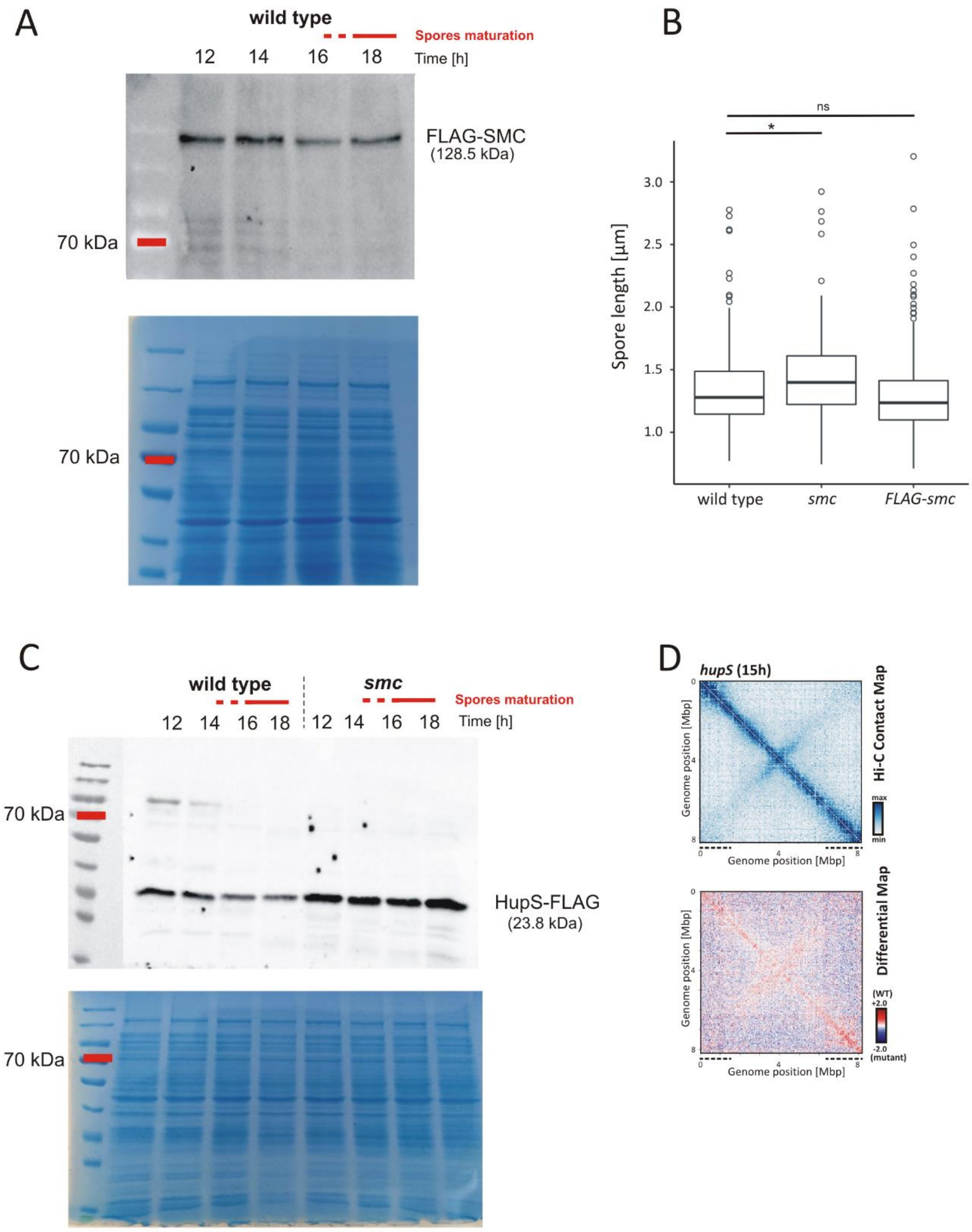
A
wild type
Spores maturation
12 14 16 18 Time [h]
FLAG-SMC
(128.5 kDa)
70 kDa
70 kDa
B
ns
*
Spore length [µm]
3.0
2.5
2.0
1.5
1.0
wild type
smc
FLAG-smc
C
wild type
smc
Spores maturation
12 14 16 18 12 14 16 18 Time [h]
70 kDa
HupS-FLAG
(23.8 kDa)
70 kDa
D
hupS (15h)
Hi-C Contact Map
Genome position [Mbp]
max
min
Differential Map
(WT) +2.0
-2.0 (mutant)

Figure S10

Supplementary Figure 10. HupS and SMC levels during sporulation. (A) Western blot detection of FLAG-SMC using anti-FLAG monoclonal antibody (dilution factor 1:1000) in the wild type background (TM017) growing for 12-18 h in MYM liquid (5 ml culture). The sporulation phase was marked with the red line. The sample processing controls is shown in the bottom panel. **(B)** Box plot analysis of the spore length distribution in the *S. venezuelae* wild type (223 spores), *smc* (173 spores) and *FLAG-smc* (293 spores) (AK200, TM010, TM017, respectively). Boxplots show median with first and third quartile while the lower and upper "whiskers" extend to the value no further than 1.5 * IQR (interquartile range) from the “hinge”. The statistical significance between strains determined by a one-way anova with a Games-Howell post-hoc test (two-sided) is marked with asterisks: p value ≤0.05 (*), ≤0.01 (**) and ≤0.001 (***). p-values: wild type-*smc*: 0.0215, wild type-*FLAG-smc*: 0.132**. (C)** Western blot detection of HupS-FLAG using anti-FLAG monoclonal antibody (dilution factor 1:1000) in the wild type background (TM015) and *smc* mutant (TM016) (growing for 14-20h in MYM liquid (5 ml culture). The sample processing control is shown in the bottom panel, The sporulation phase was marked with the red line. **(D)** The normalized Hi-C contact maps obtained for the wild type and *hupS* mutant growing for 15 h (5 ml culture). The differential Hi-C map in the logarithmic scale (log2) comparing the contact enrichment in the wild type strain (red) versus the mutant strain (blue) is shown below.

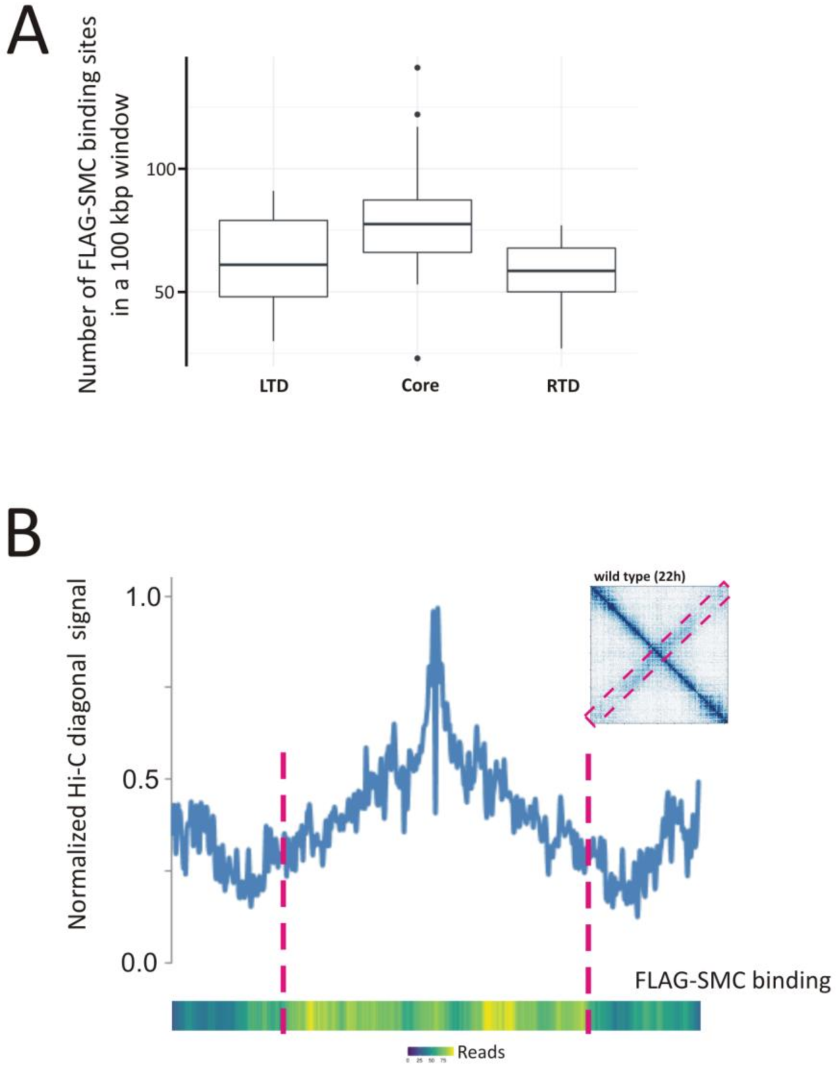

Figure S11

Supplementary Figure 11. Overlapping SMC-FLAG binding sites with the Hi-C diagonal signal. (A) Boxplot analysis of the number of SMC-bound regions (250 bp) counted in 0.1 Mbp window for core (1.9-6.3 Mbp, n = 44), LTD (<1.9 Mbp, n = 19) and RTD (>6.3 Mbp, n =

20) domains. Boxplots show median with first and third quartile while the lower and upper "whiskers" extend to the value no further than 1.5 * IQR (interquartile range) from the “hinge”. **(B)** The average value of the signal along the secondary diagonal axis was calculated using Fiji software based on the Hi-C contact map obtained for the wild type strain (*ftsZ-ypet* derivative, MD100), as shown in the insert at the 22 h of growth, and compared with the heat map of identified SMC-FLAG binding sites in the wild type background. The heat scale corresponds to the number of binding sites in the 0.5 Mbp sliding window every 1000 bp. The positions of the LTD and RTD domains are marked with black dotted lines. The pink dotted lines show the edges of LTD and RTD for easier comparison with Hi-C and ChIP-Seq data.

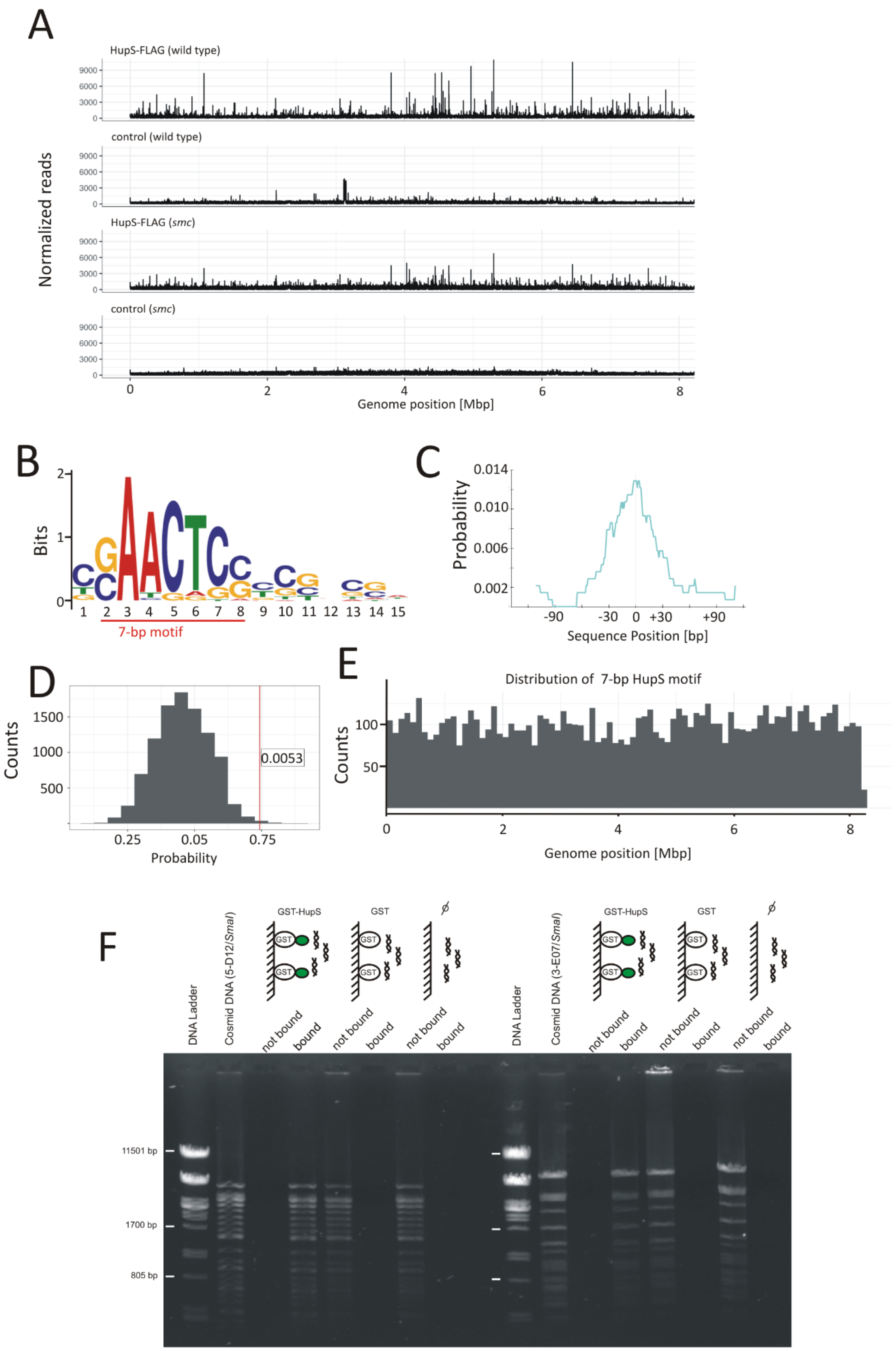
A
HupS-FLAG (wild type)
control (wild type)
HupS-FLAG (smc)
control (smc)
Normalized reads
9000
6000
3000
0
Genome position [Mbp]
B
Bits
7-bp motif
C
Probability
Sequence Position [bp]
-90
-30
0
+30
+90
D
Counts
0.0053
Probability
E
Distribution of 7-bp HupS motif
Counts
Genome position [Mbp]
F
GST-HupS
GST
DNA Ladder
Cosmid DNA (5-D12/SmaI)
Cosmid DNA (3-E07/SmaI)
not bound
bound
11501 bp
1700 bp
805 bp

Figure S12

Supplementary Figure 12. Analysis of HupS-FLAG binding. (A) The normalized ChIP-Seq reads for HupS-FLAG in the wild type background (TM015) and in the *smc* deletion background (TM016). Each experiment was supplemented with the control showing read distribution in the negative control strains of the wild type and *hupS* mutant (lacking *hupS-flag* gene). (**B**) Logo of the 15 bp sequence found by the *MEME suite* to be significantly enriched in HupS ChIP-identified regions, with the most conserved 7 bp motif indicated by the red line. (**C**) Local enrichment of probable HupS motifs in the centre of ChIP-identified regions. Plot produced by *CentriMo* software. (**D**) Permutation test showing enrichment of consensus sequences identified by the *MEME suite* in HupS ChIP-identified regions (red line, with calculated p-value shown) versus the random sequences (histogram). (**E**) Distribution of 7-bp motifs identified by the *MEME suite* across the entire S*. venezuelae* chromosome, counted in 0.1 Mbp windows. **(F)** Analysis of DNA binding by GST-HupS recombinant protein *in vitro* was performed in n = 2 independent experimental repeats. The lysates from *E. coli* BL21 pLys overexpressing GST-HupS recombinant protein (or GST protein in the control experiment) were incubated with Glutathione Sepharose resin (GE Healthcare), washed to remove unbound proteins and subsequently incubated with cosmids (5-D12, containing multiple HupS consensus sequences, and 3-E07 lacking these sequences) digested earlier with SmaI restriction enzyme. The unbound and bound (eluted with 2 M NaCl) fractions were analysed in a 1% agarose gel and visualized against DNA ladder (phage lambda DNA digested with PstI restriction enzyme) with ethidium bromide staining. As an additional control, Glutathione Sepharose resin was not incubated with *E. coli* cell lysates but only with digested cosmids.

Supplementary Table 1. *Streptomyces venezuelae* strains used in the study

**Short name/strain number**	**Relevant genotype and characteristics**	**source**
wt / -	Wild type S. *venezuelae*<br><br>NRRL B-65442 (number in NRRL culture collection , genome *NZ_CP018074.1*)	Kind gift from prof. Mark Buttner, John Innes Centre, Norwich, UK [1]
ftsZ-ypet / **MD100**	*attBΦC31*:: pKF351 *ftsZ-ypet -apra* (Apr^R)	[2]
parB (*ftsZ-ypet*) / **MD021**	*ΔparB::apra, attBΦC31::* pKF351 *ftsZ-ypet hyg* (Hyg^R , Apr^R)	[2]
parA (*ftsZ-ypet*) / **MD011**	*ΔparA, attBΦC31:: pKF351 ftsZ-ypet hyg* (Hyg^R)	[2]
- / **TM001**	*Δsmc::apra* (Apr^R),	This study
smc / **TM010**	*Δsmc::scar* (modified TM001)	This study
hupS / **AKO200**	*ΔhupS*::*apra* (Apr^R)	This study
hupS smc / **TM003**	*Δsmc::scar, ΔhupS*::*apra* (modified TM010) (Apr^R)	This study
smc (*ftsZ-ypet*) / **TM004**	Δ*smc::scar, attBΦC31*:: pKF351 *ftsZ-ypet apra* (modified TM010) (Apr^R)	This study
hupS (*ftsZ-ypet*) / **TM005**	Δ*hupS::apra, attBΦC31*:: pKF351 *ftsZ-ypet hyg* (modified AKO200) (Hyg^R)	This study
hupS smc (*ftsZ-ypet*) / **TM006**	Δ*smc::scar*, Δ*hupS::apra*, *attBΦC31*:: pKF351 *ftsZ-ypet-hyg* (modified TM003) (Hyg^R , Apr^R)	This study
wt (*ftsZ-ypet hupA-mcherry*) / **TM011**	*attBΦC31*:: pKF351 *ftsZ-ypet apra, ttBφBT1*::pSS172*hupA-mchery* (modified MD100) (Apr^R, Hyg^R)	This study
smc (*ftsZ-ypet hupA -mcherry*) / **TM012**	Δ*smc::scar*, *attBΦC31*:: pKF351 *ftsZ-ypet apra*, *attBφBT1*::pSS172*hupA -mcherry,* (modified TM010) (Apr^R, Hyg^R)	This study
hupS (*ftsZ-ypet hupA-mcherry*) / **TM013**	Δ*hupS::apra*, *attBΦC31*:: pKF351- *ftsZ-ypet* spec, *attBφBT1*::pSS172*hupA-mcherry* (modified AKO200) (Apr^R, Hyg^R, Spec^R)	This study
hupS smc (*ftsZ-ypet hupA-mcherry*) / **TM014**	Δ*smc::scar*, *attBΦC31*:: pKF351 *ftsZ-ypet spec*, *attBφBT1*::pSS172 *hupA-mcherry* (modified TM003) (Apr^R, Hyg^R, Spec^R)	This study
hupS-FLAG / **TM015**	Δ*hupS::apra, attBφBT1*:: pIJ10770-*hupS-FLAG* (modified AKO200)(Apr^R, Hyg^R)	This study
hupS-FLAG smc / **TM016**	Δ*hupS::apra*, Δ*smc::scar*, *attBφBT1*::pSS172 *hupS-FLAG* (modified	This study

	TM003) (Hyg^R, Apr^R)	
FLAG-smc / **TM017**	*smc* :: *FLAG-smc*	This study
hupS FLAG-smc / **TM018**	*smc* :: *FLAG-smc, ΔhupS::apra* (modified TM017) (Apr^R)	This study
parB FLAG-smc / **KP4F4**	*smc* :: *FLAG-smc, ΔparB*::*apra,* modified TM017) (Apr^R)	This study
- / **TM019**	Δ*smc::scar, attBφBT1*::pMS83-*smc-FLAG*. (modified TM010) (Hyg^R)	This study

Supplementary Table 2. *E. coli* strains used in the study

Strain	Relevant genotype and characteristics	Source
DH5α	*F-, Φ80dlacZΔM15, recA1, endA1, gyrA96, thi-E1, hsdR17, (rk-,mk+), supE44, relA1, deoR, Δ(lacZYA-argF)U169*	Laboratory stock
BW25113/pIJ790	(*araD-aarB*)567, Δ*lacZ*4787(::*rrnB*-4), *lacIp*-40000(*lacIQ*), λ-, *rpoS*369(*Am*), *rph-1*, Δ(*rhaD-rhaB*)568, *hsd*R514;, pIJ790 [*ori*R101], [*repA*1001(ts)], *araBp-gam-be-exo*	Laboratory stock[3]
ET12567/pUZ8002	*dam, dcm, hsdS,*CmR, TetR, pUZ8002*: tra,* KanR*, RP4 23;*	Laboratory stock[4]

Supplementary Table 3. Plasmids and cosmids used in the study

Plasmid/Cosmid	Relevant genotype and characteristics	Source
pGEM-T-Easy	Amp^R	Promega
pIJ773	pBluescript KS(+) derivative, *oriT* (RK2), FRT, (Amp^R, Apr^R)	[3]
pIJ10700	pBluescript KS(+), *oriT*(RK2), FRT (Hyg^R)	[3]
pCP20	pSC101 derivative, Rep101(Ts) *flp* (Cm^R Amp^R)	[3]
pKF351 *ftsZ-ypet apra*	pIJ6902 derivative, $attB_{\Phi C31}$, *ftsZ-ypet* under the control of the native p_{ftsZ} promoter (Apr^R)	[2]
pKF351 *ftsZ-ypet hyg*	pIJ6902 derivative, $attB_{\Phi C31}$, *ftsZ-ypet* under the control of the native p_{ftsZ} promoter (Hyg^R)	[2]
pKF351 *ftsZ-ypet*	pIJ6902 derivative, $attB_{\Phi C31}$, *ftsZ-ypet*	[2]

spec	under the control of the native p$_{ftsZ}$ promoter Spec^R	
pIJ10770	pMS82[5] derivative, intergrative vector (*attB*$_{\Phi BT1}$)(Hyg^R)	Kind gift from dr S. Schlimpert, John Innes Centre, Norwich, UK)[6]
pSS172	pMS82[5] derivative, intergrative vector (*attB*$_{\Phi BT1}$) (Hyg^R), *hupA-mcherry* under the control of the native p$_{hupA}$ promoter (Hyg^R)	Kind gift from dr S. Schlimpert, John Innes Centre, Norwich, UK)[6]
pIJ10770-*FLAG*	pIJ10770 derivative (*attB*$_{\Phi BT1}$) *FLAG* (Hyg^R)	This study
pMS83	pMS81 derivative, intergrative vector (*attB*$_{\Phi BT1}$) (Hyg^R)	[5]
pMS83-*smc*-FLAG	pMS83 derivative, *smc-FLAG* under the controla of native p$_{smc}$ promoter (Hyg^R)	This study
pIJ10770-*hupS-FLAG*	pIJ10770 derivative (*attB*$_{\Phi BT1}$) *hupS-FLAG* under the control of the native p$_{hupS}$ promoter (Hyg^R)	This study
Sv-3-B07	Cosmid (SuperCos-1) that contains *S. venezuelae* chromosomal fragment encompasing *smc* gene (*vnz_26075*) (Amp^R, Kan^R)	Kind gift from prof. Mark Buttner, John Innes Centre, Norwich, UK)
Sv-5-D08	Cosmid (SuperCos-1) that contains *S. venezuelae* chromosomal fragment encompasing *hupS* gene (*vnz_25950*) (Amp^R, Kan^R)	Kind gift from prof. Mark Buttner, John Innes Centre, Norwich, UK)
Sv-4-A09 ΔparB::apra	Cosmid (SuperCos-1) Δ*parB:: apra-oriT* (Amp^R, Kan^R)	[2]
Sv-3-B07 Δ*smc::apra*	Cosmid Sv-3-B07 Δ*smc:: apra-oriT* (Amp^R, Kan^R, Apr^R)	This study
Sv-3-B07 Δ*smc::scar*	Cosmid Sv-3-B07 Δ*smc*::scar (Amp^R, Kan^R)	This study
Sv-3-B07 Δ*smc::scar* (Apr^R)	Cosmid Sv-3-B07 Δ*smc*::scar, *bla*::*apra-oriT* (Apr^R, Kan^R)	This study
Sv-3-B07 *apra-oriT-FLAG-smc*	Cosmid Sv-3-B07 *apra-oriT-FLAG::smc* (Amp^R, Kan^R, Apr^R)	This study
Sv-3-B07 *FLAG-smc*	Cosmid Sv-3-B07 *smc:: FLAG-smc* (Amp^R, Kan^R)	This study
Sv-3-B07 *FLAG-smc* (Apr^R)	Cosmid Sv-3-B07 *smc:: FLAG-smc, bla*:: *apra-oriT* (Apr^R, Kan^R)	This study
Sv-5-D08ΔhupS*::apra*	Cosmid Sv-5-D08 □*hupS*::*apra-oriT* (Amp^R, Kan^R, Apr^R)	This study

Supplementary Table 4. Oligonucleotides used in the study

Primer	Sequence
p_{blaP1}	AATCTAAAGTATATATGAGTAAACTTGGTCTGACAGTTATGTAGGCTGGAGCTGCTTC
p_{blaP2}	CCCTGATAAATGCTTCAATAATATTGAAAAAGGAAGAGTATTCCGGGGATCCGTCGACC
p_{smc_Fw}	GGCAAGTCCAACGTCGTGGACGCCCTCTCCTGGGTCATGCATATGATTCCGGGGATCCGTCGACC
p_{smc_Rv}	GGGTTCAACACTTGAAGCAATGGGGCATGCCCGGCCTCACATATGTGTAGGCTGGAGCTGCTTC
p_{hupS_Fw}	ACGGTACCCATATGATTCCGGGGATCCGTCG
p_{hupS_Rw}	ACGAATTCCTTGTCATCGTCATCCTTGTAATCGATGTCATGATCTTTATAATCACCGTCATGGTCTTTGTAGTCCATATGTGTAGGCTGGAGCTG
$p_{apra\_FLAG\_Rv}$	ACGAATTCCTTGTCATCGTCATCCTTGTAATCGATGTCATGATCTTTATAATCACCGTCATGGTCTTTGTAGTCCATATGTGTAGGCTGGAGCTG
$p_{apra\_FLAG\_Fw}$	ACGGTACCCATATGATTCCGGGGATCCGTCG
$p_{sv\_smc\_spr\_Fw}$	CGCCGTTCCCCTCGTGTCC
$p_{sv\_smc\_spr\_rv}$	CCTTCAAGTTTCGAAGTCAATACC
$p_{hupSspr\_Fw}$	CAGGAACTCCGCAGCGGATCT
$p_{hupSspr\_Rv}$	CGGATCACCCTGGTGACGCTC
$p_{ftszypet\_fw}$	GCGGCCTTTGACTCCCTGC
$p_{ftszypet\_Rv}$	CCATCTCCGGCGGCAGCG
$P_{pSS170\_xhoI\_FLAG\_Fw}$	AGCTCTCGAGGACTACAAAGACCATGACG
$P_{pSS170\text{-}eco32I\_FLAG\_Rv}$	CCAAGCTGATATCGAATTCGTAATCATGTCATAG
$P_{pSS170\_hupS\_FLAG\_Fw}$	AGCTGGTACCACCGGTTGATGAAGGACCTCGACGAGGGC
$P_{hupS\text{-}tocherry\text{-}Rv}$	CCAAGCTGATATCGAATTCGTAATCATGTCATAG
$P_{pSS\_spr\_Fw}$	TTACCTCGCCTCTGACCCCTG
$P_{pSS\_spr\_Rv}$	GGTTCATGTGCAGCTCCATCAGC
$p_{smc\_promoter\_Fw}$	AGCTGGTACCCACGCTCCGACACCGGCC
$P_{smc\_promoter\_Rv}$	AGTCATGCATCATATGCCCGGGGACTCTACCGGG
$p_{smc\_NFLAG\_Rv}$	CCGCGGAGGGTCAGGGCCTTGAGGTGCACCTTGTCATCGTCATCCTTGTAATCGATGTC
p_{gyr2_Fd}	GCTCCGCTATCACAAGATCA
p_{gyr2_Rv}	ACAGGAAGGTCAGCAGCAG
p_{garg3_Fd}	CACCTGCGGATCTACAAGC
p_{garg3_Rv}	CCACTCCGACATCTCCTTG

$p_{smc\_poczatek\_Fw}$	AGCTCCATGGCATATGGTGCACCTCAAGGCCCTGACCCTCCGC
$p_{smc\_exp\_Rv}$	AGCTGGATCCCCGCGCAGCCGCTGGCTGATCAC

Supplementary References

1. Bibb, M. J., Domonkos, A., Chandra, G. & Buttner, M. J. Expression of the chaplin and rodlin hydrophobic sheath proteins in Streptomyces venezuelae is controlled by σ(BldN) and a cognate anti-sigma factor, RsbN. *Molecular microbiology* **84**, 1033–1049 (2012).
2. Donczew, M. *et al.* ParA and ParB coordinate chromosome segregation with cell elongation and division during Streptomyces sporulation. *Open biology* **6**, 150263 (2016).
3. Gust, B. *et al.* Red-Mediated Genetic Manipulation of Antibiotic-Producing Streptomyces. *Adances in Applied Microbiology* **54**, 107–128 (2004).
4. Kieser, T., Bibb, M. J., Buttner, M. J., Chater, K. F. & Hopwood, D. A. Practical Streptomyces Genetics. *John Innes Centre Ltd.* 529 (2000) doi:10.4016/28481.01.
5. Gregory, M. A., Till, R. & Smith, M. C. M. Integration Site for Streptomyces Phage [illegible] BT1 and Development of Site-Specific Integrating Vectors. *Journal of bacteriology* **185**, 5320–5323 (2003).
6. Schlimpert, S. *et al.* Two dynamin-like proteins stabilize FtsZ rings during Streptomyces sporulation. *Proceedings of the National Academy of Sciences of the United States of America* **114**, E6176–E6183 (2017).
