# Supplementary material for: Spatial rearrangement of the Streptomyces venezuelae linear chromosome during sporogenic development
Source: Nat Commun. 2021 Sep 1;12:5222. doi: 10.1038/s41467-021-25461-2 (PMC8410768; doi:10.1038/s41467-021-25461-2)
Supplement: Supplementary file 3 — Description of Additional Supplementary Files [file 41467_2021_25461_MOESM3_ESM.pdf]

Description of Additional Supplementary Files

File Name: Supplementary Movie 1
Description: Time-lapse observations of the wild type (*ftsZ-ypet*, *hupA-mCherry* derivative) TM011 strain. Nucleoid condensation was visualized using mCherry-HupA fusion, whereas Z-rings were visualized using FtsZ-YPet fusion. The images were taken every 10 minutes. Scale bar: 5 µm.

File Name: Supplementary Movie 2
Description: Time-lapse observations of the *hupS* mutant (*ftsZ-ypet*, *hupA-mCherry* derivative) TM013 strain. Nucleoid condensation was visualized using mCherry-HupA fusion, whereas Z-rings were visualized using FtsZ-YPet fusion. The images were taken every 10 minutes. Scale bar: 5 µm.

File Name: Supplementary Movie 3
Description: Time-lapse observations of the *smc* mutant (*ftsZ-ypet*, *hupA-mCherry* derivative) TM012 strain. Nucleoid condensation was visualized using mCherry-HupA fusion, whereas Z-rings were visualized using FtsZ-YPet fusion. The images were taken every 10 minutes. Scale bar: 5 µm.

File Name: Supplementary Movie 4
Description: Time-lapse observations of the *hupS smc* double mutant (*ftsZ-ypet*, *hupA-mCherry* derivative) TM014 strain. Nucleoid condensation was visualized using mCherry-HupA fusion, whereas Z-rings were visualized using FtsZ-YPet fusion. The images were taken every 10 minutes. Scale bar: 5 µm.
