# Supplementary material for: Spatial rearrangement of the Streptomyces venezuelae linear chromosome during sporogenic development
Source: Nat Commun. 2021 Sep 1;12:5222. doi: 10.1038/s41467-021-25461-2 (PMC8410768; doi:10.1038/s41467-021-25461-2)
Supplement: Supplementary file 8 — Reporting Summary [file 41467_2021_25461_MOESM8_ESM.pdf]

nature research

Corresponding author(s): Marcin Szafran, Dagmara Jakimowicz

Last updated by author(s): 22.07.2021

Reporting Summary

Nature Research wishes to improve the reproducibility of the work that we publish. This form provides structure for consistency and transparency in reporting. For further information on Nature Research policies, see our Editorial Policies and the Editorial Policy Checklist.

Statistics

For all statistical analyses, confirm that the following items are present in the figure legend, table legend, main text, or Methods section.

n/a	Confirmed	
☐	☒	The exact sample size (n) for each experimental group/condition, given as a discrete number and unit of measurement
☐	☒	A statement on whether measurements were taken from distinct samples or whether the same sample was measured repeatedly
☐	☒	The statistical test(s) used AND whether they are one- or two-sided<br>*Only common tests should be described solely by name; describe more complex techniques in the Methods section.*
☒	☐	A description of all covariates tested
☐	☒	A description of any assumptions or corrections, such as tests of normality and adjustment for multiple comparisons
☐	☒	A full description of the statistical parameters including central tendency (e.g. means) or other basic estimates (e.g. regression coefficient) AND variation (e.g. standard deviation) or associated estimates of uncertainty (e.g. confidence intervals)
☐	☒	For null hypothesis testing, the test statistic (e.g. F, t, r) with confidence intervals, effect sizes, degrees of freedom and P value noted<br>*Give P values as exact values whenever suitable.*
☒	☐	For Bayesian analysis, information on the choice of priors and Markov chain Monte Carlo settings
☒	☐	For hierarchical and complex designs, identification of the appropriate level for tests and full reporting of outcomes
☒	☐	Estimates of effect sizes (e.g. Cohen's d, Pearson's r), indicating how they were calculated

Our web collection on statistics for biologists contains articles on many of the points above.

Software and code

Policy information about availability of computer code

Data collection

ChIPSeq (provided by Fasteris):
HupS-ChIPseq:
- HiSeq Control Software HD 3.4.0.38
- RTA 2.7.7
- bcl2fastq2.17 v2.17.1.14

SMC-ChIPseq
- NovaSeq Control Software 1.6.0
- RTA v3.4.4
- bcl2fastq2.20 v2.20.0.422

HiC sequencing (provided by Fasteris): NextSeq Control Software 4.0.1.41; RTA 2.11.3; bcl2fastq2.17 v2.17.1.14

microscopy:Resolve3D softWoRx-Acquire Version: 6.1.1 Release 5, ImageJ 1.53c, Axio Vision Rel software

growth curve analysis: BioScreener 3.0.0

Data analysis

software for ChIP-seq data analysis: bowtie2 2.3.5.1, samtools 1.10, R software: edgeR 3.30.3, csaw 1.22.1, normr 1.14.0, rGADEM 2.36, MACS2 2.2.7.1, MEME 5.1.0, CentriMo
R software for microscopy data analysis: R Peaks 0.2, dplyr 1.0.2, ggplot2 3.3.2, findpeaks 0.0.0.9, Github: https://github.com/astrzalka/findpeaks

Hi-C data pre-processing: Trimmomatic v0.36.5, PRINSEQ v0.20.4
Hi-C data mapping: Bowtie2 v 2.3.4.3

Hi-C data processing: hicBuildMatrix v 2.1.4.0, hicMergeMatrixBins v3.3.1.0, hicCorrectMatrix v3.3.1.0, hicCorrectMatrix v3.3.1.0, hicCompareMatrixes v3.4.3.0, hicPlotMatrix v3.4.3.0, hicNormalize v3.4.3.0, hicCorrelate v3.6+galaxy0, hicPCA v3.6+galaxy0

hiCPlotMatrix v3.4.30, hiCCompareMatrices v3.4.3.0

Fiji (Image J2.0.0)

For manuscripts utilizing custom algorithms or software that are central to the research but not yet described in published literature, software must be made available to editors and reviewers. We strongly encourage code deposition in a community repository (e.g. GitHub). See the Nature Research guidelines for submitting code & software for further information.

Data

Policy information about availability of data

All manuscripts must include a data availability statement. This statement should provide the following information, where applicable:

- Accession codes, unique identifiers, or web links for publicly available datasets
- A list of figures that have associated raw data
- A description of any restrictions on data availability

The Hi-C data generated in this study (shown in Figures: 1B-D, 3A, S6A-C and S10C) have been deposited in the ArrayExpress database (EMBL-EBI) under accession code E-MTAB-9810 [https://www.ebi.ac.uk/arrayexpress/experiments/E-MTAB-9810/].
The raw ChIP-Seq data as well as the processed data generated in this study (shown in Figure 3C) have been deposited in the ArrayExpress database (EMBL-EBI) under accession code E-MTAB-9821 [https://www.ebi.ac.uk/arrayexpress/experiments/E-MTAB-9821/].
The raw ChIP-Seq data as well as the processed data generated in this study (shown in Figure 3B) have been deposited in the ArrayExpress database (EMBL-EBI) under accession code E-MTAB-9822 [https://www.ebi.ac.uk/arrayexpress/experiments/E-MTAB-9822/].

Field-specific reporting

Please select the one below that is the best fit for your research. If you are not sure, read the appropriate sections before making your selection.

☒ Life sciences ☐ Behavioural & social sciences ☐ Ecological, evolutionary & environmental sciences

For a reference copy of the document with all sections, see nature.com/documents/nr-reporting-summary-flat.pdf

Life sciences study design

All studies must disclose on these points even when the disclosure is negative.

Sample size	The sample size for all microscopy experiments was determined based on our protocols for statistical data analysis, also used in earlier studies (Kois-Ostrowska et al. 2016, Donczew et al., 2016). The sample size is determined by experiment limitation and potential to provide reliable data. The growth curves, ChIP seq and HiC experiments were performed in 3 biological replicates, as indicated in manuscript.
Data exclusions	In microscopy analysis only the hyphae which remained in the field of view and in focus throughout the whole time-lapse experiment were analysed.
Replication	The microscopy data were collected from at least 3 independent experiments (biological replicates). The ChIP seq data were collected from 2 or 3 independent experiments, and HiC data from 2-4 independent experiments. Only succesful experiments that delivered data were regarded as replicates.
Randomization	not applicable (since studies analysed different bacterial strains not population subgroups)
Blinding	When possible images were analysed using automated, non-manual image analysis methods.

Reporting for specific materials, systems and methods

We require information from authors about some types of materials, experimental systems and methods used in many studies. Here, indicate whether each material, system or method listed is relevant to your study. If you are not sure if a list item applies to your research, read the appropriate section before selecting a response.

Materials & experimental systems

n/a	Involved in the study	
☐	☒	Antibodies
☒	☐	Eukaryotic cell lines
☒	☐	Palaeontology and archaeology
☒	☐	Animals and other organisms
☒	☐	Human research participants
☒	☐	Clinical data
☒	☐	Dual use research of concern

Methods

n/a	Involved in the study	
☐	☒	ChIP-seq
☒	☐	Flow cytometry
☒	☐	MRI-based neuroimaging

Antibodies

Antibodies used

Mouse monoclonal ANTI-FLAG® M2 for Western blotting (Merck F3165-5MG) and Anti-FLAG® M2 Magnetic Beads for ChIP-seq (M8823-5ML). The dilution of the Merck F3165-5MG antibody is specified in the figure S10 legend.

Validation

Antibodies are validated by manufacturer (Merck F3165-5MG https://www.sigmaaldrich.com/specification-sheets/120/274/F3165-BULK.pdf and M8823-5ML, https://www.sigmaaldrich.com/specification-sheets/161/015/M8823-1ML_________SIGMA____.pdf)) and were tested using Western blotting and the strains expressing FLAG fusion proteins (Fig. S10).

ChIP-seq

Data deposition

☒ Confirm that both raw and final processed data have been deposited in a public database such as GEO.

☒ Confirm that you have deposited or provided access to graph files (e.g. BED files) for the called peaks.

Data access links
May remain private before publication.

Username: Reviewer_E-MTAB-9821
Password: qzjgwwgt

Username: Reviewer_E-MTAB-9822
Password: VvgYjq3p

The raw data are deposited, the bed files wil be added to databases as soon as possible.

Files in database submission

E-MTAB-9822:

sample file
dparB14_1 200928_A00902_B_L001_AOKB-149_R1.fastq.gz
dparB14_1 200928_A00902_B_L001_AOKB-149_R2.fastq.gz
dparB14_2 200928_A00902_B_L002_AOKB-168_R1.fastq.gz
dparB14_2 200928_A00902_B_L002_AOKB-168_R2.fastq.gz
dparB14_IT_1 200928_A00902_B_L002_AOKB-163_R1.fastq.gz
dparB14_IT_1 200928_A00902_B_L002_AOKB-163_R2.fastq.gz
dparB14_IT_2 200928_A00902_B_L002_AOKB-170_R1.fastq.gz
dparB14_IT_2 200928_A00902_B_L002_AOKB-170_R2.fastq.gz
dparBsmc14_1 200928_A00902_B_L001_AOKB-150_R1.fastq.gz
dparBsmc14_1 200928_A00902_B_L001_AOKB-150_R2.fastq.gz
dparBsmc14_2 200928_A00902_B_L001_AOKB-151_R1.fastq.gz
dparBsmc14_2 200928_A00902_B_L001_AOKB-151_R2.fastq.gz
dparBsmc14_3 200928_A00902_B_L001_AOKB-152_R1.fastq.gz
dparBsmc14_3 200928_A00902_B_L001_AOKB-152_R2.fastq.gz
dparBsmc14_IT_1 200928_A00902_B_L002_AOKB-164_R1.fastq.gz
dparBsmc14_IT_1 200928_A00902_B_L002_AOKB-164_R2.fastq.gz
dparBsmc14_IT_2 200928_A00902_B_L002_AOKB-165_R1.fastq.gz
dparBsmc14_IT_2 200928_A00902_B_L002_AOKB-165_R2.fastq.gz
dparBsmc14_IT_3 200928_A00902_B_L002_AOKB-166_R1.fastq.gz
dparBsmc14_IT_3 200928_A00902_B_L002_AOKB-166_R2.fastq.gz
smc14_1 200928_A00902_B_L001_AOKB-146_R1.fastq.gz
smc14_1 200928_A00902_B_L001_AOKB-146_R2.fastq.gz
smc14_2 200928_A00902_B_L001_AOKB-147_R1.fastq.gz
smc14_2 200928_A00902_B_L001_AOKB-147_R2.fastq.gz
smc14_3 200928_A00902_B_L001_AOKB-148_R1.fastq.gz
smc14_3 200928_A00902_B_L001_AOKB-148_R2.fastq.gz
smc14_IT_1 200928_A00902_B_L002_AOKB-160_R1.fastq.gz
smc14_IT_1 200928_A00902_B_L002_AOKB-160_R2.fastq.gz
smc14_IT_2 200928_A00902_B_L002_AOKB-161_R1.fastq.gz
smc14_IT_2 200928_A00902_B_L002_AOKB-161_R2.fastq.gz

smc14_IT_3 200928_A00902_B_L002_AOKB-162_R1.fastq.gz
smc14_IT_3 200928_A00902_B_L002_AOKB-162_R2.fastq.gz
wt14_1 200928_A00902_B_L001_AOKB-141_R1.fastq.gz
wt14_1 200928_A00902_B_L001_AOKB-141_R2.fastq.gz
wt14_2 200928_A00902_B_L001_AOKB-142_R1.fastq.gz
wt14_2 200928_A00902_B_L001_AOKB-142_R2.fastq.gz
wt14_IT_1 200928_A00902_B_L002_AOKB-154_R1.fastq.gz
wt14_IT_1 200928_A00902_B_L002_AOKB-154_R2.fastq.gz
wt14_IT_2 200928_A00902_B_L002_AOKB-155_R1.fastq.gz
wt14_IT_2 200928_A00902_B_L002_AOKB-155_R2.fastq.gz

E-MTAB-9821:

sample file
dsmc14_1 190517_SNK268_A_L004_AOKB-68_R1.fastq.gz
dsmc14_1 190517_SNK268_A_L004_AOKB-68_R2.fastq.gz
dsmc14_2 190517_SNK268_A_L004_AOKB-69_R1.fastq.gz
dsmc14_2 190517_SNK268_A_L004_AOKB-69_R2.fastq.gz
dsmchupS14_1 190517_SNK268_A_L004_AOKB-77_R1.fastq.gz
dsmchupS14_1 190517_SNK268_A_L004_AOKB-77_R2.fastq.gz
dsmchupS14_2 190517_SNK268_A_L004_AOKB-78_R1.fastq.gz
dsmchupS14_2 190517_SNK268_A_L004_AOKB-78_R2.fastq.gz
hupS14_1 190517_SNK268_A_L004_AOKB-72_R1.fastq.gz
hupS14_1 190517_SNK268_A_L004_AOKB-72_R2.fastq.gz
hupS14_2 190517_SNK268_A_L004_AOKB-73_R1.fastq.gz
hupS14_2 190517_SNK268_A_L004_AOKB-73_R2.fastq.gz
hupS14_3 190517_SNK268_A_L004_AOKB-74_R1.fastq.gz
hupS14_3 190517_SNK268_A_L004_AOKB-74_R2.fastq.gz
wt14_1 190517_SNK268_A_L004_AOKB-64_R1.fastq.gz
wt14_1 190517_SNK268_A_L004_AOKB-64_R2.fastq.gz
wt14_2 190517_SNK268_A_L004_AOKB-65_R1.fastq.gz
wt14_2 190517_SNK268_A_L004_AOKB-65_R2.fastq.gz

Genome browser session
(e.g. UCSC)

no longer applicable

Methodology

Replicates

Number of replicates:
HupS-chipseq

WT_14 - 2
HupS_14 - 3
dSMC_14 - 2
dSMC_HupS_14 - 2

SMC - chipseq

WT_14 - 2
SMC_14 - 3
dparB_14 - 2
dparB_SMC_14 - 3

All replicates are biological replicates.

Sequencing depth

total number / uniquely mapped reads

HupS-chipseq

WT_14 - 16259417/15763504, 13500057/13231405
HupS_14 - 16640582/15959982, 14964759/14334742, 14105646/13559757
dSMC_14 - 15903165/15438792, 17993547/17489727
dSMC_HupS_14 - 14359064/13889522, 20590375/20197098

read length 150 bp, paired-end

SMC - chipseq

WT_14 - 21141861/18059161, 19814697/17294267
SMC_14 - 22868286/16970555, 16306276/9196739, 18924060/11801043
dparB_14 - 17740243/15120009, 15515231/13251558
dparB_SMC_14 - 19917975/17613465, 17554863/15091915, 23284283/20608918

read length 75 bp, paired-end

Antibodies	mouse monoclonal ANTI-FLAG® M2 and Anti-FLAG® M2 Magnetic Beads (Merck F3165-5MG and M8823-5ML)
Peak calling parameters	HupS-chipseq<br><br>bowtie2 --local<br><br>edgeR<br>width = 69 pz<br>spacing = 23 pz<br>filter type = 'local'<br>bin length for filter = 2000 pz<br>minimal quality = 30<br>used glmQLFTest function with mergeWindows (merge distance = 100 pz, maximum peak lentgh = 5000 pz), combineTests and getBestTest functions<br><br>control files:<br>for HupS_14 - WT_14<br>for dSMC_HupS_14 - dSMC_14<br><br>MACS2 --broad<br><br>SMC-chipseq<br><br>bowtie2 default parameters<br>bam files were merged using samtools<br><br>normr<br>binsize = 250<br>mapq = 30<br>midpoint = TRUE<br><br>control - input file for each sample<br><br>WT_14 - WT_14_IT<br>SMC_14 - SMC_14_IT<br>dparB_14 - dparB_14_IT<br>dparB_SMC_14 - dParB_SMC_14_IT
Data quality	Read files were checked using fastqc.<br><br>HupS-chipseq - followed established protocol. Peaks below logFC < 2 calculated for 2000 pz surrounding region were removed from analysis. p-values were adjusted for multiple comparisons by edgeR functions: combineTests and getBestTest. Data was independently confirmed using MACS2 program.<br>Peaks belowe FDR = 0.05, logFC > 2 (all found peaks)<br>HupS_14: 142 (307)<br>dSMC_HupS_14: 61 (272)<br><br>SMC-chipseq<br><br>merged bam files were normalized and compared to input files using enrichR function from normr package. Regions found in control strains lacking FLAG protein (WT_14, dparB_14) were removed from the analysis.<br><br>Regions below FDR = 0.05, each region is 250 bp long<br>SMC_14 - 5754<br>dparB - 91<br><br>normr does not calculate logFC values for each region
Software	data collection (from Fasteris)<br>HupS-ChIPseq<br>- HiSeq Control Software HD 3.4.0.38<br>- RTA 2.7.7<br>- bcl2fastq2.17 v2.17.1.14<br>SMC-ChIPseq<br>- NovaSeq Control Software 1.6.0<br>- RTA v3.4.4<br>- bcl2fastq2.20 v2.20.0.422<br><br>bowtie2 2.3.5.1<br>samtools 1.10<br><br>R:<br>edgeR 3.30.3<br>csaw 1.22.1

normr 1.14.0
rGADEM 2.36

MACS2 2.2.7.1
MEME 5.1.0
